# Ghana’s National Health Insurance Scheme: a national level investigation of members’ perceptions of service provision

**DOI:** 10.1186/1472-698X-13-35

**Published:** 2013-08-23

**Authors:** Jenna Dixon, Eric Y Tenkorang, Isaac Luginaah

**Affiliations:** 1 Department of Geography, The University of Western Ontario, 1151 Richmond Street, London, ON N6A 5C2, Canada; 2 Department of Sociology, Memorial University, St. John’s, NF A1C 5S7, Canada

**Keywords:** Health care, Ghana, Health insurance, National health insurance scheme, Perceptions, Service

## Abstract

**Background:**

Ghana’s National Health Insurance Scheme (NHIS), established into law in 2003 and implemented in 2005 as a ‘pro-poor’ method of health financing, has made great progress in enrolling members of the general population. While many studies have focused on predictors of enrolment this study offers a novel analysis of NHIS members’ perceptions of service provision at the national level.

**Methods:**

Using data from the 2008 Ghana Demographic Health Survey we analyzed the perceptions of service provision as indicated by members enrolled in the NHIS at the time of the survey (n = 3468; m = 1422; f = 2046). Ordinal Logistic Regression was applied to examine the relationship between perceptions of service provision and theoretically relevant socioeconomic and demographic variables.

**Results:**

Results demonstrate that wealth, gender and ethnicity all play a role in influencing members’ perceptions of NHIS service provision, distinctive from its influence on enrolment. Notably, although wealth predicted enrolment in other studies, our study found that compared to the poorest men and uneducated women, wealthy men and educated women were less likely to perceive their service provision as better/same (more likely to report it was worse). Wealth was not an important factor for women, suggesting that household gender dynamics supersede household wealth status in influencing perceptions. As well, when compared to Akan women, women from all other ethnic groups were about half as likely to perceive the service provision to be better/same.

**Conclusions:**

Findings of this study suggest there is an important difference between originally enrolling in the NHIS because one believes it is potentially beneficial, and using the NHIS and perceiving it to be of benefit. We conclude that understanding the nature of this relationship is essential for Ghana’s NHIS to ensure its longevity and meet its pro-poor mandate. As national health insurance systems are a relatively new phenomenon in sub-Saharan Africa little is known about their long term viability; understanding user perceptions of service provision is an important piece of that puzzle.

## Background

Financing health care costs through health insurance schemes are a crucial tool for developing countries to avoid financial barriers to health care and protect individuals from catastrophic health care spending. Generally, those covered by insurance are more likely to have access to care and less likely to be burdened by health care costs [1]. Ghana is generally believed to have made progress in the implementation of a national level health insurance program and has often been touted as a good example for many other developing countries, including those in Africa that are looking to implement a health insurance program [2,3]. Largely subsidized through government investment and value added taxes, Ghana’s National Health Insurance Scheme (NHIS) asks for modest annual premium payments from its members, and many citizens are exempt from any payment at all. The scheme received the national government’s assent in 2003 and began operation in 2005, though exact dates vary by district.

Before the NHIS came into operation, Ghana’s health care ran on a model commonly referred to as *cash-and-carry*, which required individuals to make out-of-pocket payments at point of service usage [4,5]. Cash-and-carry, however, was wildly unpopular and socially regressive. This is largely because the poor and other marginalized populations had very little ability to access mainstream health care, and the model created disincentives to provide care in remote rural areas of the country. Ghana’s health care system during this era was chronically under staffed, under stocked and generally failed to meet the basic health needs of the populace that it set out to address in the first place [5-9]. Thus, the implementation of the NHIS in 2003 was directly purported to deal with the shortcomings of the cash-and-carry system. During the 2000 general election in Ghana, the New Patriotic Party (NPP) campaigned, and subsequently won, on a platform that promised to get rid of cash-and-carry and introduce a *pro-poor* health insurance model for the country [4].

The National Health Insurance Scheme (NHIS) was established under Act 650 of 2003 by the Government of Ghana, and technically requires all citizens to be enrolled in some form of health insurance coverage, be it private coverage, the NHIS coverage, or a combination of the two. However, in practice there is no enforcement of this rule and many citizens are without health insurance coverage of any kind [10]. These individuals must therefore pay out-of-pocket should they need treatment at a health facility, meaning that the cash-and-carry system still lives on in some form. This has resulted in a system with multiple payers – NHIS, private insurance and cash-and-carry – and a potential for hierarchies of prioritizing care. NHIS cardholders may only receive coverage at NHIS accredited facilities, though these facilities are owned and managed by different players including the government, private ownership, mission ownership and quasi-government ownership (see Table 1) [11]. While an NHIS member may be able to access the same health facility as someone paying with private insurance or paying out-of-pocket, we do not know if they are treated by the health facilities in a different manner. For instance, media reports suggest that those paying in cash get pushed to the front of the line making NHIS members wait hours for treatment [12]. Additionally, there are many private health facilities that do not recognize NHIS payments, and therefore are not accessible to NHIS members. Anecdotal accounts amongst Ghanaians recognize these facilities as higher end, especially as government facilities suffer from staffing shortages or strikes [13]. The implications of these multiple sources of health financing and multiple facilities providing service – the public’s perception of service provision via the NHIS – have yet to be explored in the literature.

**Table 1:** Ownership of NHIA accredited health facilities in Ghana

**Facility ownership**	**Number of accredited facilities as of 2010**
Government Ownership	1,460 (55.2%)
Private Ownership	1,022 (38.6%)
Mission Ownership	149 (5.6%)
Quasi-Government Ownership	16 (0.6%)
*Total*	*2,647*

Source: *NHIA* National Health Insurance Authority Annual Report 2010.

Despite these open questions, Ghana’s NHIS is in many ways regarded to have come into its own since its establishment in 2003 [14]. Previously, emphasis has been accorded to informing the population about the benefits of the scheme while encouraging people to join. The National Health Insurance Authority (NHIA), along with frequent endorsement from the World Bank, boasts an impressively high level of national enrollment starting with 1.3 million people in 2005 and increasing to 14.5 million people by 2009 (or 62% of the population) and a reported 18 million in 2010 [11,15]. However, studies suggest that enrolment varies largely by region, gender, ethnicity, and wealth, amongst other important factors [16]. Further, it has been suggested that the NHIA’s calculations for the active membership (i.e., the portion of registered members with up-to-date premium payments and card renewals) overestimate active membership by improperly tracking membership identification cards. A report by Oxfam International suggests that “the official figures used by Ghana’s NHIA are exaggerated, highly inaccurate and misleading” [17]. The Oxfam report proposes that enrolment rates may actually be as low as 18% of the population when non-renewals are taken into account, though another study suggests the 2008 rate is about 30-40%, depending on respondent’s gender [15]. More recently, the NHIA has revised its methodology for calculating active memberships putting the 2010 national rate at 33.7% of the population (8.1 million people) [18].

Irrespective of this debate, enrolment numbers mark only one of many metrics to measure the success of this health care policy. Previous research into the NHIS has focused on the social and financial characteristics of those enrolling [16,19-21] and thus far there has been limited examination of the factors associated with the perception of services once members are enrolled. Or to say otherwise, many studies have focused on barriers to enrolment, but very few have addressed the various challenges of keeping members content with the NHIS services. Given that enrolment in Ghana’s NHIS is not mandatory, and that multiple payers and providers create a competitive terrain for the NHIS, the longevity of the scheme will rest on its ability to compete with other methods of health care financing. Yet only a few studies have even remotely touched on the question of members’ perceptions of the services provided by the NHIS.

For instance, in a study of Ghana’s Dangme West District, Bruce et al. [22] point to a dissatisfaction of insured members who perceive they are given poorer quality of care and tend to wait longer compared with those making out-of-pocket payments [23]. As well, Atinga et al.’s [24] study in two hospitals in the Upper East and Northern regions of Ghana found that the care, environment of the facility and waiting time were strong determinants of patients’ perception of quality with the healthcare delivery, though they made no distinction between those enrolled and not enrolled in the NHIS.

The most noteworthy study on this topic to date is probably from Jehu-Appiah et al. [23]. Jehu-Appiah and colleagues examined household decisions to not only enroll in the NHIS, but to renew (or not renew) membership. The study, which was conducted solely in the Central and Eastern Regions, identifies, ranks and compares perceptions of insured and uninsured households on various domains of the health insurance (such as technical quality of care, convenience of the NHIS, provider attitudes, etc.). The authors found that while previous enrolment was significantly associated with the perceptions of benefits arising from the NHIS, those that no longer maintained their memberships were also less likely to find these positive elements within the scheme. The authors suggest that “even though the previously enrolled perceive NHIS to be beneficial and convenient overall, it may be that they saw fewer advantages in NHIS than originally expected” [23]. Simply put, those that dropped out perceived lower levels of service provision with the NHIS. Jehu-Appiah and colleague’s work is an important building block for understandings perceptions regarding the NHIS, however the study is limited by its regional focus and gives little insight into respondent characteristics that would help to predict such perceptions.

Thus, previous studies make relevant contributions to the literature, but leave an important issue unaddressed; there is no systematic study at the national level in Ghana on perception of service provision that provides reasonable basis for generalizations about NHIS members as a whole. Our research fills this void by examining the perceptions of members already enrolled in the scheme regarding their evaluation of the service under the NHIS, as compared to others.

Understanding the perceptions of NHIS members is important for three primary reasons. First, since the implementation of the NHIS arose from dissatisfaction with the cash-and-carry system, it is reasonable to expect that the longevity of the scheme would come from staying attune to the populace’s needs in regards to the health care system. If the alternative forms of payment (such as private insurance or paying directly out-of-pocket) are perceived as more viable alternatives, the NHIS may not be able to retain its current membership numbers and the system will falter from lack of premium contributions^a^. Regardless of objective reality of the quality of service provision, members’ subjective evaluations are ultimately what will determine their participation in the NHIS. Second, there have been anecdotal reports that new methods of health facility payment via the health insurance authority may result in cash flow problems and thus a decline in patient care [10]. Thus, members’ perception of service provision may be the first indication of these problems.

Third, while national level health insurance schemes have been researched extensively in developed countries, they are relatively new endeavors in developing countries such as Ghana. Noting that numerous sub-Saharan countries including Rwanda, Burkina Faso, Nigeria, Kenya and South Africa have either started a national health insurance program or are in the advanced stages of starting one, the insights from Ghana may be helpful in their developments. We attempt to fill this important gap by examining the predictors of members’ perceptions of service provision using the most recent version of Ghana Demographic and Health Survey. The novelty of this research is therefore beneficial not only for further policy development in Ghana, but potentially also for other health insurance schemes starting up around the continent.

## Methods

### Data and sample

We used data from the 2008 Ghana Demographic and Health Survey (GDHS), which is made free and publically available upon registration with the DHS (http://www.measuredhs.com). The Ghana Demographic and Health survey (GDHS) is a nationally representative data set administered by Ghana Statistical Service and Macro, and the fifth in such surveys of the Global Demographic and Health Surveys Program. The GDHS provides high quality and reliable quantitative data on basic demographic and health indicators and quite recently has introduced a module on the National Health Insurance Scheme (NHIS). The GDHS identified about 5096 eligible women aged 15–49 from 11,778 households out of which 4916 were interviewed, yielding a response rate of 97%. Approximately 4769 eligible men aged 15–59 were also identified with 4568 interviewed from the same household resulting in a response rate of 96%. The analysis for this study was however restricted to 1422 males and 2046 females who are already enrolled in the NHIS.

### Measures

The dependent variable for this study ‘perception of NHIS service provision’ was measured with the question: “In your opinion, do NHIS card holders get better/same/worse service than others?” This question was only asked to respondents who indicated they had health insurance and then specified “National/District Health Insurance” as the type of health insurance coverage. Although the literature on the perceptions of health insurance in developing countries is thus far quite scant, available studies show economic, socio-cultural and demographic variables as important correlates of enrolment in health insurance [16,19-21,25,26]. Taking cues from these studies, we examine the effects of socio-economic variables (wealth and education) and socio-demographic and cultural variables (age, ethnicity, marital status, place of residence, and region of residence) on perception of service provision with the NHIS in Ghana. The wealth quintile, which we employ as a socio-economic variable, was constructed from weighted scores on household ownership of consumer items and dwelling characteristics. This was coded as ‘Poorest’, ‘Poorer’, ‘Middle’, ‘Richer’, and ‘Richest’. The educational background of respondents is also coded as ‘no education’, ‘primary education’ and ‘secondary/higher education’. Other theoretically relevant socio-demographic variables include marital status (never married, currently married, formerly married), age of respondent, religious affiliation (Christians, Muslims, Traditionalists, and no religion), ethnicity (Akan, Ga Dangbe, Ewe, Northern languages and other), urban or rural residence, and finally region of residence (Southern Ghana or Northern Ghana). Northern, Upper East, and Upper West regions were classified as Northern Ghana. Greater Accra, Central Western, Brong Ahafo, Volta, Eastern and Ashanti regions were grouped as Southern Ghana.

### Analytical strategy

The Ordinal Logistic Regression technique was applied to examine the relationship between perception of NHIS service provision with coverage and socioeconomic and demographic characteristics. This method is appropriate given that the response variable employed for analysis is polytomous and has a natural ordering to it. Of the three possible responses (worse, same or better service), “worse” was used as the reference category. Thus, an odds ratio greater than one indicates a greater likelihood of saying the health insurance scheme service is the same or better, than to say it is worse. On the contrary, an odds ratio less than one indicates a lower likelihood of reporting that the health insurance scheme service is the same or better, than to say it is worse.

## Results

Univariate results in Table 2 are provided for the dependent variable and selected explanatory variables for the sample of male (*n* = 1422) and female (*n* = 2046) respondents who were card-carrying members of the NHIS. The majority of male respondents (55.3%) and a high percentage of female respondents (46.9%) identified their level of service with the NHIS as better. Respectively, about 34.2% and 43.2% of males and females rated their level of service as the same. Finally around one tenth of respondents felt that their level of service was worse (10.5% of males and 9.9% of females).

**Table 2:** Univariate results for select independent and dependent variables

**Perception of NHIS service provision**	**Males (n = 1422)**	**Females (n = 2046)**
	**% of respondents**	**% of respondents**
Worse	10.5	9.9
Same	34.2	43.2
Better	55.3	46.9
**Wealth quintile**		
Poorest	15.2	16.8
Poorer	15.4	15.9
Middle	15.8	17.8
Richer	26.3	24.0
Richest	27.3	24.7
**Education**		
No Education	11.1	20.5
Primary Education	11.8	16.8
Secondary/Higher education	77.0	62.7
**Marital Status**		
Never married	41.2	29.8
Currently married	56.1	63.0
Formerly married	2.7	7.3
**Age**	32.2 (15–59)	29.3 (range: 15–49)
**Religious affiliation**		
Christians	72.3	76.8
Muslims	20.2	18.0
Traditionalists	4.4	2.9
No religion/other	3.1	2.3
**Ethnicity**		
Akans	43.1	46.3
Ga Dangbe	5.1	5.3
Ewe	11.1	9.3
Northern	36.7	36.0
Other	4.0	3.0
**Rural–urban residence**		
Urban	49.8	48.3
Rural	50.2	51.7
**Region of residence**		
Southern Ghana	67.6	69.3
Northern Ghana	32.4	30.7

Respondents were fairly evenly distributed amongst the five wealth quintiles, though there were slightly more males and females identifying as either ‘rich’ or ‘richer’. Similarly, the large majority of this sample had secondary or higher education (about 77% of males and 62.7% of females respectively. Most respondents were married at the time of the survey (56.1% of males and 63.0% of females), while 41.2% of male and 29.8% of females had never been previously married. Around three quarters of males and females identified their religious affiliation as Christian, followed by Muslims at around a fifth of the sample. A small proportion identified themselves as traditionalists. In terms of ethnicity, a high proportion of those sampled identified themselves as either Akan or of the Northern languages, followed by the Ewe or Ga Dangbe ethnicity. Males and females were split relatively even between urban and rural residency. About a third of respondents were from the North compared to two-thirds from the South.

Table 3 provides results of the bivariate relationships between the dependent variable ‘perception of NHIS service provision’ and selected independent variables. Findings suggest that wealthier Ghanaians are significantly less likely to report that their perceived NHIS service was the same or better than to report it was worse. This relationship holds true for both males and females, though is stronger for the male respondents. Similar observations are made for females with higher levels of education. When compared to those with no education females with secondary or higher education were 38% less likely to indicate that their perception of NHIS service was the same or better, than to report it was worse.

**Table 3:** Bivariate analysis of selected dependent and independent variables

**Independent variables**	**Perception of NHIS service provision**	
**Wealth quintile**	**Males (n = 1422)**	**Females (n = 2046)**
Poorest (ref)	1.00	1.00
Poorer	.971 (.220)	.998 (.177)
Middle	.692 (.168)	.768 (.123)*
Richer	.560 (.122)***	.746 (.121)*
Richest	.450 (.096)***	.764 (.124)*
**Education**		
No Education	1.00	1.00
Primary Education	1.01 (.229)	.868 (.132)
Secondary/Higher education	.765 (.133)	.721 (.092)***
**Marital Status**		
Never married	1.00	1.00
Currently married	.976 (.106)	1.15 (.111)
Formerly married	.598 (.176)*	.890 (.153)
**Age**	1.01 (.003)	.999 (.004)
**Religious affiliation**		
Christians	1.00	1.00
Muslims	1.69 (.293)***	1.01 (.157)
Traditionalists	1.05 (.255)	1.81 (.563)**
No religion	1.03 (.325)	1.03 (.328)
**Ethnicity**		
Akans	1.00	1.00
Ga Dangbe	1.32 (.409)	.583 (.125)***
Ewe	1.04 (.212)	.685 (.124)**
Northern	1.74 (.255)***	1.10 (.145)
Other	1.03 (.378)	.540 (.145)**
**Rural–urban residence**		
Urban	1.00	1.00
Rural	1.48 (.206)***	1.24 (.155)*
**Region of residence**		
Southern Ghana	1.00	1.00
Northern Ghana	1.68 (.272)***	1.30 (.185)*

Note: ***p < .01; **p < .05;*p < .1.

Odds ratios are adjusted for clustering and robust standard errors are presented in brackets. Northern, Upper East and Upper West regions are classified as ‘Northern Ghana’, while Greater Accra, Central, Western, Brong Ahafo, Volta, Eastern and Ashanti regions are grouped as ‘Southern Ghana’.

Bivariate results also indicate that compared to males that were never married, formerly married males were less likely to report that their perception of NHIS services were the same or better, than worse when compared to the previous cash-and-carry model and other existing alternatives. Regarding religion, Muslim males and female Traditionalists were significantly less likely to report that the NHIS was same or better, than to report it was worse, compared with Christians. Males who identified their ethnicity as ‘Northern ethnic group’ were 74% more likely to report that they perceived NHIS cardholders received same or better service than worse service when compared to males of Akan ethnicity. Similarly, females of Ga Dangbe and other ethnicities were about half as likely to rate their perception of service as same or better than to rate the service as worse, compared to females of Akan ethnicity. The rural–urban and regional divide is particularly notable at the bivariate level. Respectively, both males and females in rural areas were significantly more likely (48% and 24%) to perceive that NHIS cardholders received same or better service than worse service, compared to those in urban areas. Similarly, when compared to males and females in the Southern regions of the country, those from the Northern region were significantly more likely to perceive cardholders received same or better service than worse.

Mulitvariate models are presented in Table 4. Three main models each are built for both males and females. In the first model, we examine the relationship between socioeconomic variables (wealth and educational background) on perception of NHIS service provision. The second model adds sociodemographic variables including marital status, age, religious affiliation, ethnicity, and rural/urban residence. Finally, the third model controls region of residence, which we have earlier identified as crucial for NHIS enrolment.

**Table 4:** Odds for perception of NHIS service provision to be ‘same’ or ‘better’ among men and women in Ghana, 2008

**Independent variables**	**Men**			**Women**		
**Wealth quintile**	**Model 1**	**Model 2**	**Model 3**	**Model 1**	**Model 2**	**Model 3**
Poorest (ref)	1.00	1.00	1.00	1.00	1.00	1.00
Poorer	.945 (.222)	.921 (.236)	.940 (.244)	1.05 (.190)	1.07 (.201)	1.10 (.215)
Middle	.674 (.169)	.635 (.179)	.681 (.200)	.841 (.139)	.872 (.150)	.942 (.177)
Richer	.538 (.126)***	.522 (.142)***	.578 (.184)*	.832 (.145)	.835 (.153)	.922 (.190)
Richest	.429 (.097)***	.434 (.113)***	.489 (.152)**	.871 (.156)	.904 (.142)	1.01 (.233)
**Education**						
No Education	1.00	1.00	1.00	1.00	1.00	1.00
Primary Education	1.17 (.275)	1.21 (.287)	1.23 (.309)	.895 (.139)	.880 (.142)	.869 (.144)
Secondary/Higher						
education	1.13 (.216)	1.26 (.242)	1.25 (.250)	.778 (.107)*	.761 (.107)**	.759 (.114)*
**Marital Status**						
Never married		1.00	1.00		1.00	1.00
Currently married		-	.841 (.156)		-	1.18 (.161)
Formerly married		-	.562 (.184)*		-	.972 (.203)
**Age**		-	1.01 (.006)		-	.992 (.006)
**Religious affiliation**						
Christians		1.00	1.00		1.00	1.00
Muslims		1.49 (.321)*	1.53 (.321)**		1.01 (.171)	1.01 (.172)
Traditionalists		.687 (.215)	.664 (.210)		1.42 (.460)	1.41 (.463)
No religion		.834 (.269)	.860 (.280)		.872 (.275)	.869 (.272)
**Ethnicity**						
Akans		1.00	1.00		1.00	1.00
Ga Dangbe		1.26 (.374)	1.24 (.363)		.555 (.119)***	.550 (.117)***
Ewe		1.01 (.218)	.987 (.212)		.647 (.118)**	.638 (.117)***
Northern		1.23 (.222)	1.16 (.286)		.927 (.150)	.791 (.160)
Other		.801 (.343)	.778 (.320)		.484 (.138)***	.464 (.135)***
**Rural–urban residence**						
Urban		1.00	1.00		1.00	1.00
Rural		-	1.14 (.202)		-	1.09 (.176)
**Region of residence**						
Southern Ghana		-	1.00		-	1.00
Northern Ghana		-	1.09 (.320)		-	1.22 (.280)
**Log Pseudo-likelihood**	**−1323.8245**	**−1313.0039**	**−1310.7385**	**−1927.9633**	**−1914.0935**	**−1911.1388**
**Model significance (Wald**						
**Chi-sq)**	23.31 (5)***	33.51 (13)***	41.11(18)***	**9.54 (6)**	30.19 (13)***	36.82 (18)***
**Pseudo R2**	**.014**	**.021**	**.023**	**.001**	**.01**	**.01**
**Threshold 1**	**−2.52**	**−2.34**	**−2.05**	**−2.49**	**−2.62**	**−2.66**
**Threshold 2**	**-.567**	**-.373**	**-.074**	**-.158**	**-.267**	**-.298**

Note: ***p < .01;**p < .05;*p < .1.

Odds ratios are adjusted for clustering and robust standard errors are presented in brackets. Northern, Upper East and Upper West regions are classified as ‘Northern Ghana’, while Greater Accra, Central, Western, Brong Ahafo, Volta, Eastern and Ashanti regions are grouped as ‘Southern Ghana’.

Consistent with the bivariate results, wealth was found to be a statistically significant predictor of respondents’ perceptions regarding NHIS service, especially for men. Compared to the poorest, wealthier men are about 51% less likely to perceive that services from the NHIS were the same or better than to perceive it as worse (see Table 4, model 3). It is worth noting however that the statistical significance of wealth was attenuated when region of residence was controlled for in the final model (see models 2 and 3 in Table 4). Although wealth was not statistically associated with the perception of NHIS service provision for female respondents, education was. Compared to females with no education, those with secondary or higher education were less likely (0.759 times) to perceive the NHIS service as the same or better than to perceive it as worse. Compared to males that had never been married, males that were formerly married were almost half as likely to perceive the NHIS service as the same or better than to perceive it as worse. Ethnicity did not appear to be an important influencing factor for male respondents. However, it did appear to be a strong influence for female respondents. Compared to Akans, women of Ewe, Ga Dangbe and of other ethnic backgrounds were significantly less likely to perceive the NHIS service was the same or better.

## Discussion

Arguably, the most significant finding is the influence of wealth on perceptions of the service provided by the NHIS. We observe in particular that wealthier men were significantly less likely to perceive NHIS services as the same or better compared to perceiving the services as worse. In other words, wealthy Ghanaian men perceived lower levels of services provided by the NHIS than other financing options. Possible explanations include the fact that, although enrolled in the NHIS, wealthy Ghanaian men have alternative health financing options including purchase from private facilities or travel abroad, and thus the NHIS service would seem worse in comparison. These results are punctuated by the national media’s focus on Ghana’s now deceased President and other high-ranking public officials traveling overseas for personal medical treatment [27-29]. Alternatively, those that cannot afford to pay for health care out-of-pocket would certainly find any level of service to be better under the NHIS as they are not financially obliged to pay per use for their medical treatment, as was the case under the cash-and-carry system. Therefore the perceived level of service provision among Ghanaian men may be linked with the ability to purchase alternatives to the health care provided under the NHIS.

Notwithstanding, this finding is still intriguing considering that across the board, studies have found that the wealthy are more likely to enroll in the NHIS [16,19-21,25,26]. It is however not very clear as to why the wealthy will be more likely to enroll in the NHIS, while simultaneously perceiving the service provision to be worse than the alternatives. A potential explanation could be that the wealthier are often making a substantial contribution to the NHIS through their Social Security taxes, and this may raise their expectations above the poor. Furthermore, considering that the NHIS premiums are relatively cheap, another plausible reason is that rich males may purchase it only for a back up, and not use it as a primary source of care. This may also mean that these men have firsthand experiences being served with alternative payment options (i.e., private insurance) providing them with the opportunity to make comparisons with the NHIS. This result does, however, imply that the reports of NHIS’ cash flow issues [10] which would result in poorer patient care and those paying by cash being pushed to the front of the line may not be severe enough to impact the perception of service provision with the NHIS. Were this the case, we would expect that poor males would respond similarly to rich males, and find the NHIS to be the worse than other forms of payments. Since poor males rated their perception of NHIS service provision to be relatively high, we understand that they are receiving better care than they would be via the cash-and-carry system, the assumed alternative for the poor.

In contrast to male respondents, wealth was not significantly associated with women’s perceptions of NHIS service provision, and we suggest that this result throws light on the gender dynamics of intra-household resource allocation. Tolhurst et al. [30] reveal that in Ghana the decisions regarding healthcare are primarily about access and control over resources between household members. Commonly in Ghanaian households, women are expected to seek the male household head’s approval before they or the children spend resources on health care. Approval to spend household resources is intimately tied with a bargaining process that is influenced by norms of power and the women’s responsibility for children, norms of responsibility for payment, and marital status. During the cash-and-carry era it was not uncommon for women or children to delay seeking medical treatment due to the absence of the father or his refusal to pay for health care [30]. Though men are expected to cover health care costs, in reality women would often take on the burden these payments. If women were not receiving sufficient support from their husbands, Tolhurst and Nyonator [31] found that they would often attempt to renegotiate support by withholding co-operation on other responsibilities in the household, or ‘reporting’ the man to the elders.

It is important to point out that the wealth ranking given to female respondents is applied at the household level and does little to elucidate their actual ability to make out-of-pocket payments for health care. If a woman previously had to go through the male household head in order to access money every time that she or the children were in need of health services, then health insurance coverage could serve as a way around this bargaining process. As only one annual premium payment is required for full NHIS coverage, regardless of their wealth status women would stand to benefit from being insured by avoiding frequent bargaining and debates regarding health care payments. Or to say otherwise, women would actually be able to access care via the NHIS, which is better service than no care at all if they were relying on their husband to pay in cash up front. Thus women’s perception of service with the NHIS would have little to do with their household wealth ranking, as provided by the GHDS. Further, the status of educated women as somewhat empowered and independent or engaged in the household decision making regarding health matters likely speaks to a modern move away from these traditional intra-household bargaining experiences, and therefore would more likely reflect the tendency among males to generally report poor perceptions of the NHIS’ services, as our results have demonstrated.

Our study does however point to the strong influence of ethnicity on women’s perception of NHIS services, specifically that the Akan women are far more likely to report perceiving NHIS services to be the same or better as alternatives. Some studies suggest that quality of care impacts perceived benefit of health insurance [32,33]. There is evidence to the effect that the Akan-speaking areas, primarily the Ashanti region, have some of the best facilities and strongest usage of health services out of the entire country. For instance, in both 2008 and 2009 the Ashanti region had the second lowest doctor-to-person ratios in the country next to the Greater Accra Region and a very high hospital bed usage (see Table 5) [34]. It is possible therefore that the quality of healthcare delivery in the Akan dominated areas reflects in the high usage and positive perceptions of the NHIS services derived from such health facilities, and that these may be mutually re-enforcing. Furthermore, the reason that this relationship would show up significantly in women and not men, relates back to the culturally determined roles that either men or women are expected to fulfill. In Ghana, women tend to be more closely linked with caring for the health of the family and children [30], and would be the ones to realize the benefits of having the health insurance and usage of the health facilities.

**Table 5:** Population per Doctor and hospital bed occupancy by region, 2008 and 2009

**Region**	**Population per Doctor**		**Hospital bed occupancy**	
	**2009**	**2008**	**2009**	**2008**
**Ashanti Region**	8,288 (2)	9,537 (2)	65.5% (2)	63.3% (2)
**Brong Ahafo Region**	16,919 (4)	21,475 (4)	57.9% (5)	60.5% (4)
**Central Region**	22,877 (5)	26,140 (5)	51.4% (9)	52.5% (7)
**Eastern Region**	16,132 (3)	17,571 (3)	55.9% (6)	55.6% (6)
**Greater Accra Region**	5,103 (1)	4,959 (1)	78.3% (1)	72.0% (1)
**Northern Region**	50,751 (10)	68,817 (10)	59.1% (4)	61.3% (3)
**Upper East Region**	35,010 (8)	33,475 (8)	49.7% (10)	42.2% (10)
**Upper West Region**	40,144 (9)	43,988 (9)	62.1% (3)	59.4% (5)
**Volta Region**	26,538 (6)	27,959 (6)	54.1% (7)	51.1% (9)
**Western Region**	33,187 (7)	31,745 (7)	52.0% (8)	51.5% (8)
***National:***	*11,929*	*12,713*	*59.8%*	*58.3%*

Note: Rankings by region are presented in brackets.

*Source:* Ghana Health Service: *2009 GHS Annual Report.*

### Limitations

While this study has produced interesting findings, there are a number of limitations. First, we are unable to draw ‘causal’ relationships between the dependent and independent variables due to the cross-sectional nature of the data. Second, caution must be exercised with the interpretation of results as the GDHS’ question on members’ perception of NHIS services as the question tapped the subjective interpretations of respondents and may not reflect an objective assessment of members’ experiences with the scheme (i.e., perceived provision of services tells us little about the actual access, utilization and level of care that a respondent received). This problem characterizes most survey research where data are self-reported, and signals the need for more conceptually rigorous survey questions. Notwithstanding, the study provides important insights and raises relevant policy questions regarding members’ perception of services and, in turn, the overall sustainability of the NHIS in Ghana.

## Conclusions

This paper provides important insights into the factors associated with the ongoing viability of Ghana’s NHIS. Using a national level sample we have assessed the factors associated with perception of NHIS service provision as compared to other options and, we argue, this is a key component to its longevity. Though not technically legal, the *de facto* continuation of cash-and-carry in addition to media reports of better services via private insurance or at privately run clinics means that the NHIS faces competition on many fronts. While respondents for this study do not specify which alternative they are comparing NHIS service provision to, we argue that the strength of the NHIS depends on being competitive with all the alternatives. Given that Ghana’s move towards the NHIS was politically motivated by the public’s strong dissatisfaction with the cash-and-carry system, the focus of the current policy makers must reflect an appropriate balance of the need to optimize enrolment while minimizing opt out rate. This will not succeed if current members perceive other options as providing better service.

The results of this study stand in contrast to previous work that has solely looked at influences on enrolment on the NHIS, thus demonstrating a difference in influences between those who are enrolling in the NHIS and those who do enroll and perceive the services they receive to be comparatively worthwhile. Simply put, there is a difference between originally enrolling in the NHIS because one believes it is potentially beneficial, and alternatively, using the NHIS and finding it to truly be of benefit. For instance, while our previous study demonstrated a positive relationship between wealth and the likelihood of enrolment for men and women [16], this study shows a negative relationship between wealth and perception of NHIS service provision.

Perception of NHIS service provision differs greatly between males and females, with wealthy men less likely to perceive the service to be the same or better than the alternatives. We suggest this is based largely on the power of choice that money provides, cultural norms, gender relationships and intra-household resource allocation. This could also mean that if such men become disgruntled with the NHIS, or perceive better service via other forms of payment, they could discontinue their memberships altogether and it would be women and children who are likely to be left behind.

Further, it was proposed in this previous work that some of the differences geographically in enrolment patterns could be attributed to social networks and ‘word of mouth’ encouragement to sign up for the NHIS. Similarly, Jehu-Appiah et al. [23] found that a household’s decision to enroll is influenced by community attributes such as health beliefs, attitudes and peer pressure. If this holds true, it seems that community encouragement to join the scheme may work the first time, but does little to influence perceptions once they have already enrolled in the NHIS.

There are also other important implications that emanate from this investigation. One of the underlying tenants of a health insurance program is that a wide variety of citizens – healthy and unhealthy, rich and poor, and so on – must enroll in order to balance out the input and drain on the financial pool that sustains the system. For instance, economist often raise concerns about the problems of voluntary health insurance and the creation adverse selection, where unhealthy people who would foresee heavy use of the health care system are more interested in enrolling in the health insurance and healthy people self select out of the insurance. This, down the road, could potentially impact health care costs and contributions, and lead to the discontinuation of the health insurance program [35,36]. Unless policy makers can maintain relatively equal enrolment from all areas of the population, the NHIS will face stability problems in the future. Thus, determining and understanding what variations in perceptions towards the NHIS is the first step in being able to tailor the program to meet the needs of all Ghanaians.

## Endnotes

^a^At the time of data collection (2008) only 5% NHIS total inflow was attributed to premium payments. In contrast, a large majority of inflow (61.5%) was derived from the 2.5% tax levy, and a sizeable amount (16.9%) from Social Security contributors, who are provided exemptions from premium payments for NHIS enrolment. This low inflow from premium payments can be attributed to the large proportion of members who are exempt from making premium payments; in 2009 this constituted 70.6% of all members [15]. While this policy supports the scheme’s ‘pro-poor’ stance, the NHIS has been challenged with financial difficulties, as indicated in annual reports [18] and notably made public with the NHIA’s inability to pay outstanding bills to Mission owned facilities in 2013 [37]. Though we are not suggesting here that this financial instability has been caused by the numerous exempted users (instead corruption and internal misuse of resources are more likely culprits), nonetheless an erosion of premium paying members from the scheme would not help this situation.

## Competing interests

The authors declare that they have no competing interests.

## Authors’ contributions

JD was the primary author and completed the literature review and discussion sections, and participated in the design of the study. EYT took the lead in the design of the study, carried out the statistical analysis, created all the tables and helped in reading and proofing and manuscript drafts. IL assisted in study design, and helped in reading and proofing and manuscript drafts. All authors read and approved the final manuscript.

## Pre-publication history

The pre-publication history for this paper can be accessed here:

http://www.biomedcentral.com/1472-698X/13/35/prepub
